# Spinal Nerve Axotomy: Effects on I_h_ In Vivo and HCNs in DRG Neurons

**DOI:** 10.3390/ijms252312889

**Published:** 2024-11-30

**Authors:** Yuanlong Song, Linlin Gao

**Affiliations:** 1 Department of Physiology, School of Basic Medicine, Tongji Medical College, Huazhong University of Science and Technology, 13 Hangkong Rd., Wuhan,430030, China; songyuanlong@hust.edu.cn; 2 Hubei Key Laboratory of Drug Target Research and Pharmacodynamic Evaluation, Huazhong University of Science and Technology, 13 Hangkong Rd., Wuhan,430030, China

**Keywords:** I_h_, HCN, spinal nerve axotomy, in vivo voltage clamp

## Abstract

In vitro experiments performed on dissociated dorsal root ganglion (DRG) neurons suggest the involvement of the hyperpolarization-activated cation current (I_h_) in enhancing neuronal excitability, potentially contributing to neuropathic pain. However, the more confirmative in vivo information about how nerve injury interacts with I_h_ is lacking. In this study, I_h_ was recorded in vivo using the dynamic single-electrode voltage clamp (dSEVC) technique on L5 DRG neurons of normal rats and those seven days after spinal nerve axotomy (SNA). Compared to normal rats, SNA unexpectedly inhibited the activity of I_h_ channels on A-fiber DRG neurons: (a) the I_h_ current magnitude, density, and conductance were consistently diminished; and (b) the I_h_ activation velocity was slowed and the voltage for I_h_ activation was hyperpolarized. The half-activation voltage (V_0.5_) exhibited a negative shift, and the time constant for I_h_ activation was prolonged across all test potentials, indicating the reduced availability of I_h_ after SNA. To further investigate the mechanisms of SNA on I_h_, the underlying HCN channels and the correlated mRNA were quantified and compared. The mRNA expression level of *HCN*1-4 was uniformly enhanced after SNA, which might have contributed to the increased cytoplasmic HCN1 intensity observed in both medium- and large-sized DRG neurons. This finding contradicted the functional reduction of I_h_ after SNA. Surprisingly, the HCN labeling pattern was altered after SNA: the labeling area of HCN1 and HCN2 at the membranous ring region of the axotomized large neurons became significantly thinner or absent. We concluded that the diminished ring immunoreactivity for HCN1 and HCN2 correlated with a reduced availability of I_h_ channels, elucidating the observed decrease in I_h_ in axotomized A-fiber neurons.

## 1. Introduction

Neuropathic pain can manifest as various painful sensations following peripheral nerve injuries, which may be either spontaneous or triggered by stimuli, such as allodynia and hyperalgesia. The underlying mechanisms of neuropathic pain remain inadequately understood; however, it has been proposed that factors such as heightened afferent firing, the increased sensitivity of nociceptors (peripheral sensitization), and enhanced responses to afferent signals within the central nervous system (central sensitization) may contribute to its development [1].

The hyperpolarization-activated cation channel, I_h_, which is encoded by the *HCN*1-4 genes, is activated when the membrane potential becomes hyperpolarized. The inward current generated upon the activation of the I_h_ channel results in membrane depolarization. I_h_ is believed to play a role in maintaining the resting membrane potential, influencing the excitability of excitable cells, and increasing the firing frequency by reducing the duration of the after-hyperpolarization phase. HCN channels are extensively expressed in DRG neurons with diverse sensory properties, trigeminal ganglion neurons, and central neurons involved in sensory signal transduction. Numerous studies have indicated a close association between I_h_ and the onset and persistence of neuropathic pain; however, there appears to be inconsistency regarding the roles of the central and peripheral I_h_ in this context.

For instance, the downregulation of I_h_ expression was observed in the dendrites of the anterior cingulate cortex (ACC) following a chronic constriction injury (CCI) of the sciatic nerve. This downregulation of I_h_ enhances the synaptic transmission efficiency, thereby contributing to the hyperexcitability of ACC neurons and the manifestation of hyperalgesia behavior [2]. In line with these observations, the upregulation of I_h_ has been shown to alleviate hyperalgesia induced by CCI [3].

In the peripheral nervous system, extensive research has demonstrated that a peripheral nerve injury results in the upregulation of I_h_ and hyperexcitability in DRG neurons, characterized by a reduced rheobase, increased evoked discharge frequency, and even ectopic spontaneous firing [4,5,6,7]. Furthermore, it has been suggested that the upregulation of I_h_ may be a critical factor driving the hyperexcitability of DRG neurons, as the application of the I_h_ blocker ZD7288 has been shown to decrease the discharge frequency and hyperalgesia resulting from a peripheral nerve injury [4,7,8,9]. Thus, peripheral I_h_ appears to be a significant contributor to neuropathic pain.

However, it is noteworthy that the upregulation of I_h_ has thus far only been documented in vitro, specifically in dissociated and cultured DRG neurons, and has not been previously investigated in vivo. Our prior observations indicate that the electrophysiological properties of DRG neurons in vivo may differ substantially from those observed in vitro. Hence, we aimed to determine whether I_h_ is upregulated in vivo in axotomized L5 DRG neurons seven days post-spinal nerve axotomy (SNA). We also aimed to investigate if there are any alterations in the characteristics of I_h_ that would enhance the likelihood of hyperexcitability in DRG neurons. Given our previous findings regarding I_h_ in normal DRG neurons with established sensory properties, we compared the I_h_ in axotomized DRG neurons to that in normal, undamaged DRG neurons. This comparison aimed at providing clearer insights into how nerve injury affects the current density and activation characteristics of I_h_ in vivo. Additionally, we examined the expression levels of *HCN*1-4 mRNA and HCN1-2 protein for both sets of DRG neurons to further clarify the relationship between I_h_ and neuropathic pain resulting from a peripheral nerve injury.

## 2. Results

### 2.1. I_h_, I_h_ Density, and Kinetics

Neurons were classified based on their CVs into the categories Aα/β-, Aδ-, and C- following the loss of their receptive fields after L5 SNA. In total, I_h_ was recorded in 93 L5 DRG neurons from 14 SNA rats and compared with I_h_ in 128 L4-L6 DRG neurons from 24 normal rats. The axotomized L5 DRG neurons comprised five C-, 12 Aδ-, and 76 Aα/β-neurons, while the normal neurons included 14 C-, 18 Aδ-, and 96 Aα/β-neurons. The I_h_ properties in normal DRG neurons with established sensory characteristics were detailed in my previous publication [10].

**Figure 1:**
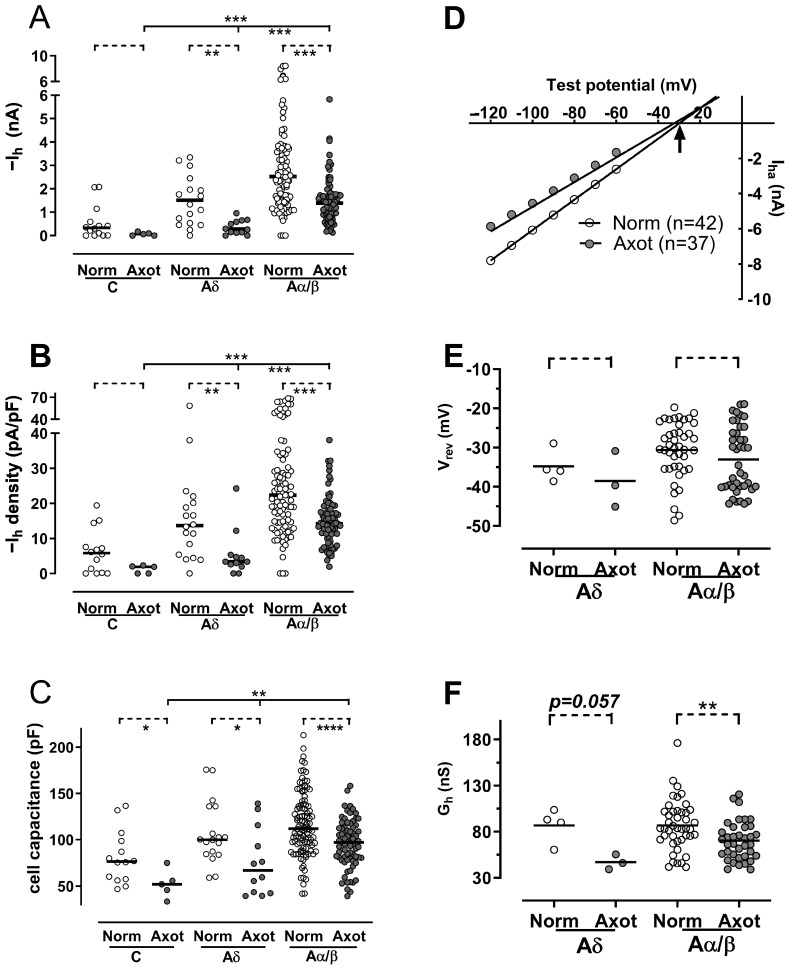
The effect of nerve axotomy on I_h_ density and properties. (**A**,**B**), I_h_ and I_h_ density vs. neuronal type; I_h_ and I_h_ density were reduced in C-, Aδ-, and Aα/β-neurons seven days after axotomy, but this reached significance only in Aδ- and Aα/β-neurons. After axotomy, the axotomized Aα/β-neurons still had significantly higher I_h_ and I_h_ density than axotomized Aδ- and C-neurons. (**C**), cell capacitance was compared between normal and axotomized neurons. It was significantly reduced in C-, Aδ-, and Aα/β-neurons after nerve axotomy. (**D**), I–V curves for normal and axotomized neurons. V_rev_s were the intersections of the I–V curves to the *X*-axis (indicated by arrows); the slope of the curve indicated I_h_ conductance (G_h_), 67 nS (median, n = 37) for axotomized vs. 84 nS (median, n = 42) for normal neurons. (**E**,**F**), scatter plots for V_rev_ and slope for normal and axotomized DRG neurons. The V_rev_ was not changed by nerve axotomy, but the G_h_ was reduced significantly in Aα/β-neurons. Dashed lines on the top of graph in this figure and figures onwards indicate Mann–Whitney tests for comparison of two variables; solid lines indicate Kruskal–Wallis tests for comparison of more than two variables. *: *p* < 0.05; **: *p* < 0.01; ***: *p* < 0.001; ****: *p* < 0.0001.

I_h_ at −100 mV in axotomized neurons was measured as described in the Section 4. Axotomized Aα/β-neurons exhibited a significantly higher I_h_ compared to Aδ- and C-neurons (*p* < 0.001 for both) (Figure 1A). In axotomized Aδ-neurons, the median I_h_ was −0.28 nA (n = 12), which represents an approximately 80% decrease compared to the median I_h_ in normal Aδ-neurons (−1.51 nA, n = 18, *p* < 0.01). Similarly, the median I_h_ in axotomized Aα/β-neurons (−1.39 nA, n = 76) decreased by 30% compared to normal Aα/β-neurons (−2.53 nA, n = 96) (*p* < 0.001). The median I_h_ in axotomized C-neurons (−0.06 nA, n = 5) was also reduced by 80% compared to normal C-neurons (−0.33 nA, n = 14), although this difference was not statistically significant (*p* = 0.06).

The median cell capacitance in axotomized C-, Aδ-, and Aα/β-neurons was 51.8 pF, 66.9 pF, and 96.9 pF, respectively (Figure 1C). In contrast, the capacitance values for normal C-, Aδ-, and Aα/β-neurons were 76.5 pF, 100.0 pF, and 111.8 pF, respectively. The axotomy-induced decrease in cell capacitance across all three CV groups was significant (*p* < 0.05 for C- and Aδ-neurons, *p* < 0.0001 for Aα/β-neurons). This reduction in cell capacitance suggests neuronal shrinkage following nerve axotomy.

The I_h_ density in axotomized neurons exhibited changes similar to those observed for I_h_ (see Figure 1B compared to Figure 1A). For instance, the median I_h_ density in axotomized Aα/β-neurons was −14.2 pA/pF (n = 73), representing a 36% decrease compared to −22.3 pA/pF in normal Aα/β-neurons (n = 95). This reduction in the I_h_ density was statistically significant across all CV groups (*p* < 0.05 for C- and Aδ-neurons, *p* < 0.0001 for Aα/β-neurons).

The reversal potential (V_rev_) and conductance (G_h_) were obtained from 37 out of 93 axotomized DRG neurons. Among the 37 axotomized neurons, there were three Aδ-neurons and 34 Aα/β-neurons, with no C-neurons present. Consequently, these axotomized neurons were compared to 42 normal A-fiber neurons, which included four Aδ-neurons and 38 Aα/β-neurons. The reversal potentials were determined as the intersections of I–V curves with the *X*-axis, yielding values of −29.9 mV for normal neurons and −32.3 mV for axotomized neurons (Figure 1D).

In both Aα/β- and Aδ-neurons, V_rev_ was not significantly altered by nerve axotomy (*p* > 0.4 for both) (Figure 1D,E). However, G_h_ was significantly decreased in axotomized Aα/β-neurons (*p* < 0.05, Mann–Whitney test). A similar trend was observed in axotomized Ad-neurons, with median values of 91.5 nS for normal neurons compared to 46.2 nS for those subjected to SNA. The lack of statistical significance (*p* = 0.057) may be attributed to the small sample size (n = 4 for normal and n = 3 for SNA) (Figure 1F).

**Figure 2:**
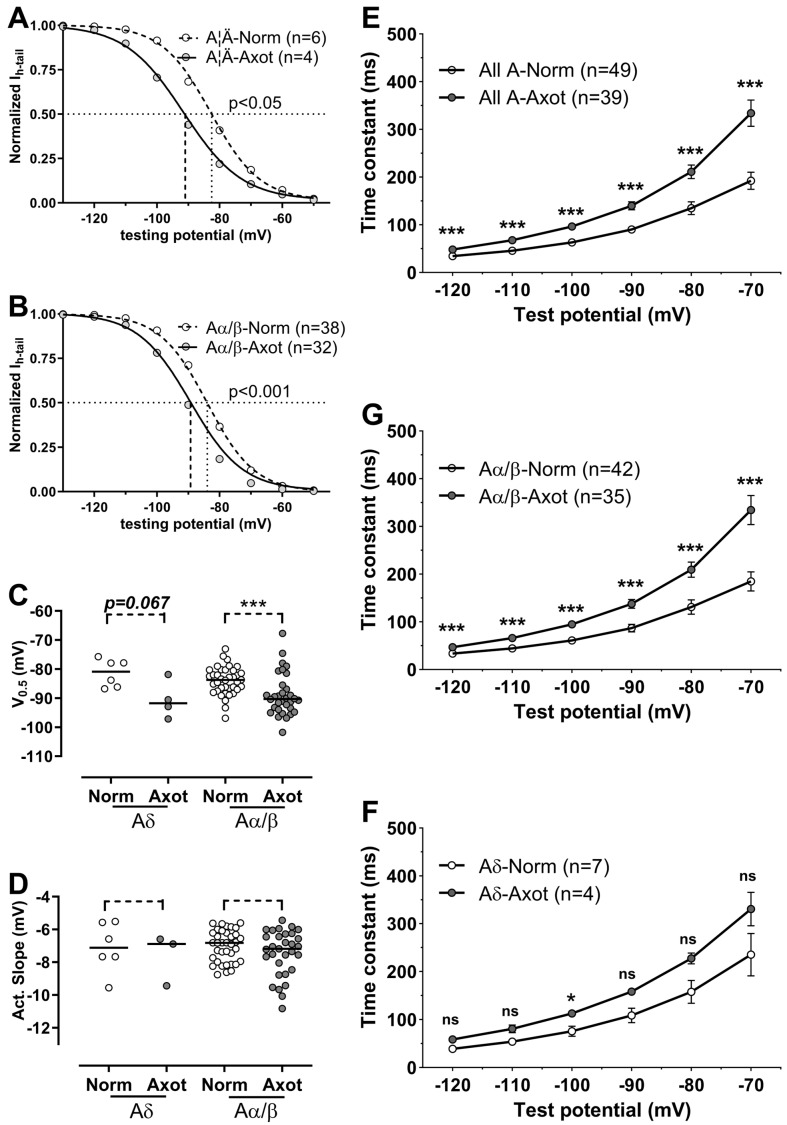
The effect of nerve axotomy on I_h_ kinetics. (**A**,**B**), activation curves for normal and axotomized Aδ- and Aα/β-neurons from which V_0.5_ and slope were derived. V_0.5_ in both axotomized Aδ- and Aα/β-neurons were more negative. (**C**,**D**), scatter plots for V_0.5_s and slopes of activation curves for normal and axotomized neurons. The slopes were unchanged, while V_0.5_ in axotomized Aδ- and Aα/β-neurons was significantly more negative. (**E**–**G**): the activation time constant of I_h_ (τ_act_) was compared in normal and axotomized A-fiber neurons. Similar to comparisons for all A-neurons, axotomized Aα/β-neurons had significantly slower τ_act_ than normal Aα/β-neurons in the whole range of test potentials (−70 mV~−120 mV). Axotomized Aδ-neurons also had slower τ_act_, although the difference was not significant. *: *p* < 0.05, ***: *p* < 0.001, ns: not significant.

The half-activation potential (V_0.5_) and slope were determined from 36 axotomized neurons (four Aδ-, 32 Aα/β-) by fitting the activation curves to the Boltzmann equation. V_0.5_ and slope were measured in two normal C-nociceptors, but not in any axotomized C-neurons. Consequently, the V_0.5_ and slope were compared with those in 42 normal A-fiber DRG neurons (six Aδ-, 36 Aα/β-). After axotomy, the steepness (slope) of the activation curve for both Aδ- and Aα/β-neurons did not change (*p* > 0.2 for both, Figure 2D). However, the median V_0.5_ in axotomized Aα/β-neurons (−90.3 mV) was significantly more negative than that in normal Aα/β-neurons (−83.8 mV, *p* < 0.001) (Figure 2B,C). The median V_0.5_ in axotomized Aδ-neurons was −91.7 mV (n = 4) and was more negative compared to −80.9 mV (n = 6) in normal Aδ-neurons (Figure 2A,C). This change was not significant (*p* = 0.067), likely due to the small sample size (n = 4)

The I_h_ activation time constant (τ_act_) was measured in 39 axotomized A-fiber neurons (4 Aδ- and 35 Aα/β-neurons) and compared with 49 normal A-fiber neurons (7 Aδ- and 42 Aα/β-neurons). The τ_act_ in axotomized A-neurons was significantly longer than that in normal A-neurons (*p* < 0.001 at all test potentials) (Figure 2E). For instance, at −100 mV, the medians for tact were 59 ms and 94 ms, respectively, in normal and axotomized A-fiber neurons. The τ_act_ was analyzed separately in Aα/β- and Aδ-neurons. Both axotomized Aα/β- and Aδ-neurons exhibited longer τ_act_; however, the change was significant only in axotomized Aα/β-neurons across all test potentials (*p* < 0.001 for all cases) (Figure 2F). In axotomized Aδ-neurons, and τ_act_ was significantly longer only at −100 mV (*p* < 0.05) (Figure 2G). The lack of significance for Aδ-neurons may be attributed to the small sample size (n = 7 for normal vs. n = 4 for axotomized).

### 2.2. HCN1-4 mRNA Levels

**Figure 3:**
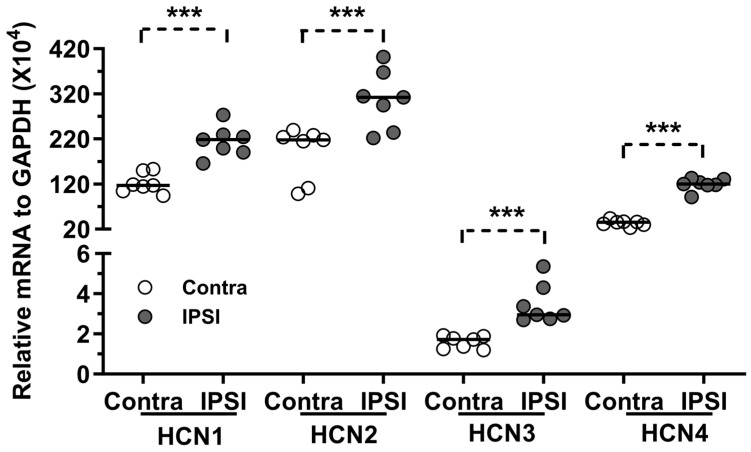
The effect of nerve axotomy on mRNA of *HCN*1-4. Quantitative RT-PCR technique was performed to test the mRNA for *HCN*1-4 in ipsi- and contra-sides of both L4 and L5 DRG neurons seven days after L5 SNA. The relative mRNA for all *HCN*1-4 was significantly increased in ipsilateral L5 DRGs. ***: *p* < 0.001.

Quantitative RT-PCR was performed to evaluate the impact of nerve axotomy on the mRNA levels of *HCN*1-4 seven days following SNA. For each rat, the mRNA levels of *HCN*1-4 in axotomized L5 DRGs were compared to those in the contralateral L5 DRGs. The results indicated that the mRNA levels for all four types of *HCN*s were significantly elevated in axotomized neurons: *HCN*1 showed a 1.9-fold increase, *HCN*2 a 1.4-fold increase, *HCN*3 a 1.7-fold increase, and *HCN*4 a 3.4-fold increase. These findings suggest an upregulation of *HCN*1-4 mRNA levels induced by peripheral nerve injury (Figure 3).

### 2.3. Pain-Related Behaviors

Spontaneous pain, tactile allodynia, cold allodynia, and thermal hyperalgesia were assessed both ipsilaterally and contralaterally on days one, three, and seven following nerve axotomy (n = 11).

No SNA rats exhibited spontaneous foot lifting during the observation periods, which aligns with previous findings [11].

**Figure 4:**
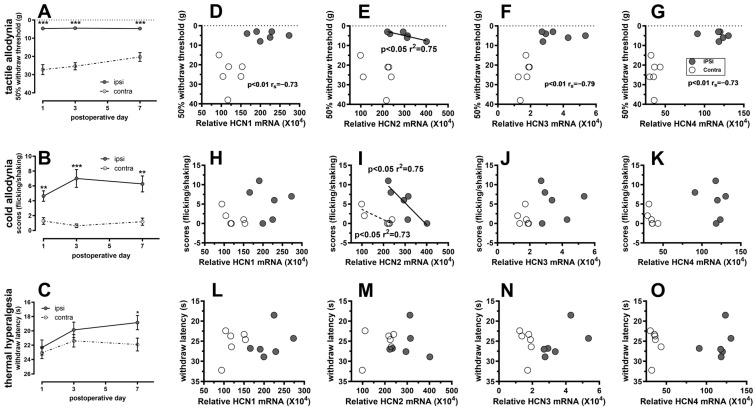
Neuropathic pain behaviors vs. mRNA for *HCN*s seven days after axotomy. (**A**), the median 50% withdrawal threshold for ipsilateral side was significantly lower than contralateral side at day one, three, and seven after SNA; (**B**), SNA rats showed significantly more ipsilateral flicking, licking, or shaking to one drop of acetone application to the plantar surface of the hind paw at day one, three, and seven after axotomy; (**C**), the hind paw on the ipsilateral side showed shorter latency of withdrawal to noxious thermal stimuli only on day seven. In all cases, *Y*-axis from the bottom to the top indicated increased pain. * *p* < 0.05, ** *p* < 0.01, *** *p* < 0.001. Both ipsilaterally (filled circle) and contralaterally (open circles), mRNA of *HCN*1-4 was plotted against pain behaviors (n = 6). The correlation of the amount of *HCN*1-4 mRNA and pain-related behaviors were examined separately for ipsilateral and contralateral side (linear regression), and for both sides together (Spearman’s regression). (**D**–**G**), *HCN*1-4 mRNA *vs*tactile allodynia: tactile allodynia was linearly correlated with ipsilateral *HCN*2 mRNA, and was negatively correlated with mRNA of *HCN*1, 3, and 4 with a Spearman coefficient of ~0.7. (**H**–**K**), *HCN*1-4 mRNA vs. cold allodynia: *HCN*2 mRNA was linearly correlated with cold allodynia both ipsilaterally and contralaterally. (**L**–**O**), *HCN*1-4 mRNA vs. thermal hyperalgesia. The *Y*-axis in all figures from the bottom to the top indicated increased pain.

In comparison to the contralateral side, the 50% withdrawal threshold to von Frey hairs was significantly reduced on day one (*p* < 0.001) following the SNA. A comparable decrease in the withdrawal threshold was also observed on days three (*p* < 0.001) and seven (*p* < 0.001) (see Figure 4A). The SNA rats exhibited a significantly heightened response, characterized by flicking, licking, or shaking, to a single drop of acetone applied to the plantar surface of the left hind paw one day post-operation (*p* < 0.01). This response to acetone was further amplified on day three after SNA (*p* < 0.001) and showed a slight recovery by day seven (*p* < 0.01) (see Figure 4B). The withdrawal latency to noxious heat, which serves as an indicator of thermal hyperalgesia, was significantly shorter on the ipsilateral side, with statistical significance noted only on day seven (*p* < 0.05) following SNA (see Figure 4C). These findings suggest that the SNA models employed in this study exhibited neuropathic pain behaviors consistent with those previously documented in the literature [7,11].

Pain-related behaviors did not exhibit a correlation with the I_h_ amplitude (data not presented) in the limited number of animals studied (n = 6).

### 2.4. The Correlation Between Pain-Related Behaviors and the mRNA Levels of HCN1-4

Pain-related behaviors were evaluated in six out of seven SNA rats, during which the levels of *HCN*1-4 mRNA were quantified. The mRNA levels were subsequently plotted against the observed pain behaviors (refer to Figure 4D–O). Notably, two distinct subgroups emerged, corresponding to the ipsilateral and contralateral sides, particularly evident in the data pertaining to tactile allodynia. On the ipsilateral side, elevated levels of *HCN*1-4 mRNA were associated with increased pain responses, whereas the contralateral side exhibited lower levels of *HCN*1-4 mRNA and reduced pain responses. A linear regression analysis was performed to evaluate the correlation between *HCN*1-4 mRNA levels and pain behaviors, with separate analyses conducted for the ipsilateral and contralateral sides. The results indicated a negative correlation between *HCN*2 mRNA levels and the severity of both tactile and cold allodynia on the ipsilateral side. Furthermore, a negative correlation was also observed between cold allodynia and *HCN*2 mRNA levels on the contralateral side. These findings suggest that decreased levels of *HCN*2 mRNA are associated with heightened tactile and cold allodynia.

When both the ipsilateral and contralateral data were aggregated, Spearman’s rank correlation analysis was utilized to evaluate the correlations. The results indicated that only tactile allodynia demonstrated a negative correlation with the mRNA levels of *HCN*1, *HCN3*, and *HCN4*.

### 2.5. HCN1 and HCN2 Immunoreactivity

Neurons were classified into three size categories: small, medium, and large, based on their cross-sectional area, which was defined as follows: small (0–400 µm^2^), medium (400–800 µm^2^), and large (>800 µm^2^). A total of 316 neurons from the ipsilateral side and 340 neurons from the contralateral side of the L5 DRGs were analyzed for ring and cytoplasmic pixel density. The cross-sectional area of neurons on the ipsilateral side ranged from 130 to 1700 µm^2^, whereas on the contralateral side, it ranged from 160 to approximately 2000 µm^2^. These findings indicate a reduction in neuronal size following nerve axotomy.

**Figure 5:**
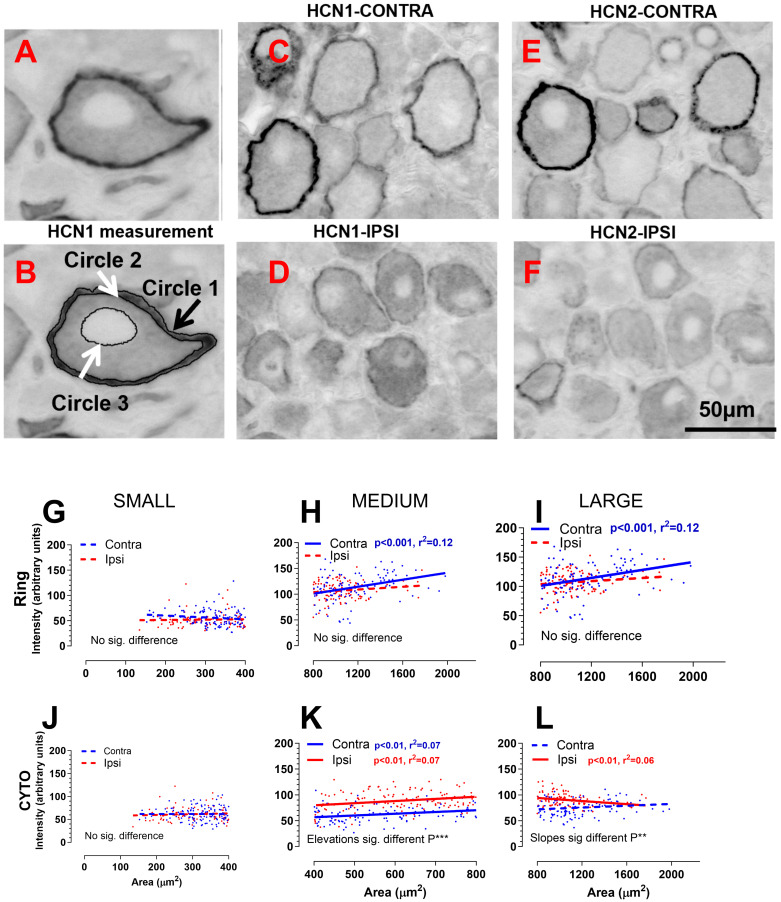
HCN1-2 immunoreactivity in DRGs seven days following SNA. (**A**,**B**), In a neuron with visible nucleus, HCN1 membrane-related (ring) and cytoplasmic immunostaining was measured using Simple PCI by drawing three circles in the neuron: circle one is over the outline of the ring, circle two is inside the ring, and circle three is around the outline of nucleus. (**C**–**F**), immunocytochemistry images for HCN1 and HCN2: the ring staining of HCN1 and HCN2 seen contralaterally became weaker or vanished ipsilaterally, but the cytoplasmic staining was higher. The scale bar in the right side of the lower layer is for all images in (**C**–**F**). (**G**–**I**), the comparison of HCN1 ring intensity separately in small (<400 μm^2^), median (400~800 μm^2^), and large (>800 μm^2^) neurons; (**J**–**L**), the comparison of HCN1 cytoplasm intensity separately in small, medium, and large neurons. The red and blue symbols and lines represent L5 DRG neurons in ipsilateral and contralateral sides, respectively. The dotted lines represent that there are no correlations between HCN1 pixel density and neuronal area, while the solid lines indicate that there is a significant linear correlation between them. Significance inside figures represents whether HCN1 pixel density differed ipsilaterally and contralaterally. **: *p* < 0.01; ***: *p* < 0.001.

In small neurons, the intensity of the HCN1 ring and cytoplasmic levels remained consistent across different neuronal areas, showing no significant differences between the ipsilateral and contralateral sides (see Figure 5G,J). In medium-sized neurons, both the HCN1 ring and cytoplasmic intensity displayed a linear correlation with the neuronal size, indicating elevated levels in neurons of larger dimensions. When comparing the ipsilateral side to contralateral L5 DRG neurons, both the HCN1 ring and cytoplasmic intensity were significantly higher on the ipsilateral side (see Figure 5H,K). In large neurons, only the intensity of the HCN1 ring exhibited a positive correlation with the neuronal size on the contralateral side, with a darker ring observed in larger contralateral L5 neurons (see Figure 5I). Axotomized large neurons demonstrated an increased HCN1 cytoplasmic intensity, but a decrease in the HCN1 ring intensity when compared to L5 DRG neurons on the contralateral side (see Figure 5I,L).

HCN1 and HCN2 immunoreactivity was observed to be both membrane-associated and cytoplasmic. Notably, the staining patterns of HCN1 and HCN2 exhibited significant differences between normal and axotomized DRG neurons (refer to Figure 5C–F). On the contralateral side, the membrane-associated immunoreactivity for HCN1 and HCN2 was markedly more pronounced than the cytoplasmic staining, resulting in a distinct ring of variable thickness. In contrast, on the ipsilateral side, the HCN1 and HCN2 rings were either thinner in certain neurons or completely absent in others, despite the cytoplasmic staining for HCN1 and HCN2 being more intense.

## 3. Discussion

This study investigated the impact of nerve axotomy on I_h_ in vivo. In contrast to previous in vitro studies, our current findings in vivo indicated a reduced I_h_ amplitude and increased difficulty in activating I_h_ following axotomy. This reduction can be attributed to decreased immunostaining for HCN1-2, which is the predominant HCN subtype found in axotomized large DRG neurons. The SNA rat model exhibited neuropathic pain behaviors consistent with those observed in analogous nerve injury models. Notably, pain behaviors did not correlate with the levels of I_h_ or the mRNA expression of *HCN*1-4, with the exception of *HCN*2.

Our observations of a reduced I_h_ density and conductance, along with more negative V_0.5_ values and slower activation kinetics post-axotomy, contradict previous studies that reported an increase in I_h_ under diabetic neuropathy conditions [12,13] and following peripheral nerve injury [4,14,15]. The differences between our in vivo findings and previous in vitro studies may be attributed to the dissociation and culture methodologies employed by the latter researchers. Our prior research has demonstrated that the electrophysiological characteristics of DRG neurons undergo significant alterations following dissociation compared to their counterparts within a living organism [16]. Additionally, several factors should be considered: (1) The present study was conducted at approximately 30 °C while the in vitro studies were performed at room temperature. (2) Different experimental models were utilized—we employed the SNA model whereas Yao et al. (2003) used the chronic compression injury (CCD) model [14] and Chaplan et al. (2003) employed the spinal nerve ligation (SNL) model [4].

Regarding the expression of *HCN*1-4 mRNA in contralateral L5 DRGs, the current findings suggest that HCN1 and HCN2 are the most prevalent isoforms in DRG neurons, followed by HCN4, while HCN3 is present at significantly lower levels (approximately one-tenth that of HCN1). This observation aligns with the previous studies conducted by Chaplan et al. [4] and Kouranova et al. [17], which also reported high levels of HCN1 and HCN2 in DRG neurons. The low expression of HCN3 in the current study is consistent with the results reported by Chaplan et al.; however, Kouranova et al. observed a considerably higher expression of HCN3, comparable to that of HCN1. This discrepancy may be attributed to differences in methodologies employed between these studies. While Chaplan et al. and the current study extracted mRNA from whole DRGs, Kouranova et al. isolated mRNA from a specific population of 100–150 DRG neurons categorized by size. It is plausible that Kouranova’s study included a small subpopulation of DRG neurons with relatively high HCN3 expression.

An elevation in mRNA levels for all *HCN*1-4 isoforms was observed in axotomized L5 DRGs compared to the contralateral L5 DRGs. In contrast, Chaplan et al. (2003) reported a decrease in the *HCN*1-4 mRNA levels in the L5/L6 DRGs on the operated side seven days after spinal nerve ligation (SNL), compared to sham-operated rats. These findings present a fundamental discrepancy with the current results. Several factors may account for this divergence: (1) The current study employed a spinal nerve axotomy (SNA) model involving the complete axotomy of the L5 spinal nerve following tight ligation, whereas Chaplan et al. utilized an SNL model that involved the tight ligation of the L5/L6 spinal nerves without axotomy. This methodological difference may have allowed for the preservation of some nerve fibers, which could be influenced by peripheral factors such as inflammation in the damaged segment of the nerve. (2) The mRNA levels in axotomized L5 DRGs were compared with those in unaffected contralateral L5 DRGs, whereas Chaplan et al.’s control DRGs from sham-operated rats may have been influenced by the surgical intervention and subsequent inflammatory responses.

Upon initial examination, it may seem contradictory that a reduction in the I_h_ current was observed alongside an increase in *HCN*1-4 mRNA and HCN1 cytoplasmic immunoreactivity. One plausible explanation for this phenomenon is the altered distribution pattern of HCN1-2 immunoreactivity in axotomized DRGs compared to the contralateral side. Based on my previous findings [10], it is likely that HCN1-2 predominantly contributes to I_h_ in A-fiber neurons, as evidenced by the strong immunostaining of HCN1-2 in medium-to-large-sized neurons. However, axotomized DRG neurons exhibited diminished or absent HCN1-2 ring immunoreactivity and significantly increased cytoplasmic HCN1-2 immunostaining. This suggests a reduction in the presence of these proteins in the neuronal membrane and/or a failure of newly synthesized HCN proteins to localize properly within the membrane. Consequently, the inadequate distribution of HCN channels to the membrane may lead to an accumulation of these proteins in the cytoplasm, resulting in lower HCN1-2 ring immunoreactivity and a corresponding decrease in the availability of functional I_h_ channels in the neuronal membrane, which accounts for the reduced I_h_ observed in axotomized A-fiber neurons.

Another potential explanation for the observed discrepancies may arise from the misclassification of DRG neurons due to significant neuronal shrinkage following spinal nerve axotomy. Immunohistochemical analyses and measurements of cell capacitance indicate that axotomized DRG neurons are generally smaller than their normal counterparts. Despite this, the same size criteria were maintained for classifying neurons into small, medium, and large categories. Consequently, it is possible that some of the larger axotomized neurons were incorrectly categorized within the medium-sized group. Notably, both normal and severed DRG neurons tend to show an increase in nucleic acids and proteins expression levels as the cell size progresses from small to large. Therefore, if larger axotomized neurons were included in the medium-sized cell group, it would artificially elevate the expression levels within this particular group.

The role of I_h_ in modulating the electrical properties of neurons, particularly their excitability, has long been a subject of investigation and remains a complex issue. I_h_ is known to be partially activated in the resting state of excitable cells, leading to an inward current that depolarizes the cell membrane and moves it closer to the action potential threshold, thereby enhancing neuronal excitability. Conversely, the expression of I_h_ can also result in a decrease in the input resistance of the cell membrane and an increase in conductance. Consequently, during excitation conduction, the presence of a local current can produce a minimal voltage change across the membrane due to the low input resistance, which impedes membrane depolarization and diminishes excitability. This phenomenon is commonly referred to as the current-conductance conundrum. Thus, it is important to consider factors such as the specific neuronal location, the level of I_h_ expression, and the presence of other ion channels nearby when assessing its influence on neuronal excitability. The current consensus suggests that I_h_ functions more as a buffering mechanism or a bidirectional regulator in most central nervous system neurons rather than exerting a unidirectional effect that solely increases or decreases the excitability.

In contrast to various observations made in smaller dorsal root ganglion (DRG) neurons [18,19,20], extensive in vivo and in vitro electrophysiological studies have demonstrated that axotomized A-fiber neurons exhibit increased excitability following peripheral nerve injury, as evidenced by a reduced rheobase and an increased frequency of evoked firing [21,22]. It has been proposed that the hyperexcitability of A-fiber DRG neurons is primarily mediated by sodium channels, although I_h_ may also play a contributory role. The application of I_h_ blockers has been shown to decrease firing frequency, and neurons exhibiting a higher level of bursting activity displayed larger I_h_ currents [22]. However, this evidence is insufficient to conclusively establish that I_h_ is the primary factor responsible for the increased firing rates in axotomized A-fiber neurons, as commercially available I_h_ blockers lack specificity for HCN channels and have been demonstrated to also inhibit calcium (C_av_), sodium (N_av_), potassium (K_v_), and human ether-a-go-go-related gene (h_ERG_) channels [23,24,25,26,27,28]. Furthermore, no significant effect of I_h_ on spontaneous firing rates was observed following an intravenous injection of the I_h_ blocker ZD7288 during the current in vivo experiments despite achieving an approximately 90% blockade of I_h_ (our unpublished observations, n = 3).

While there is still uncertainty surrounding the specificity of ZD7288 in inhibiting I_h_ and whether this inhibition genuinely reduces the firing frequency, we acknowledge that neurons exhibiting high firing rates are associated with elevated levels of I_h_ current. The latest in vivo findings suggest that axotomized A-fiber neurons exhibiting the highest firing rates correspond to muscle spindles (unpublished observations), which indeed exhibit the largest I_h_ currents [10].

Overall, the impact of I_h_ expression on neuronal excitability and the firing frequency remains ambiguous; a reduction in I_h_ does not necessarily correlate with decreased excitability in axotomized A-fiber neurons. In fact, recent research suggests that high I_h_ expression may be more likely to diminish the excitability and firing frequency of DRG neurons [29]. Consequently, a reduction in I_h_ could potentially enhance the excitability of DRG neurons, thereby contributing to the onset and persistence of neuropathic pain. This notion is further supported by a double-blind clinical trial indicating that ivabradine, an inhibitor of I_h_, did not alleviate hyperalgesia [30,31], suggesting that reducing or blocking the I_h_ current may not be an effective strategy for managing neuropathic pain.

Limitations of this study: Firstly, due to the inherent limitations of in vivo recording, it is expected that the probability of successfully recording smaller cells would be lower compared to larger cells; therefore, biases may exist when considering the distribution of data across cell sizes. Secondly, a combination of pentobarbital and pancuronium bromide muscle relaxant was used to maintain anesthesia and muscle relaxation for stable in vivo recording. Although this might potentially alter the electro properties of neurons, it did not affect the interpretation of the current data as they were compared with data obtained under similar experimental conditions. Therefore, cautions should be exercised when comparing these findings with those obtained from in vitro recordings.

## 4. Materials and Methods

**Figure 6:**
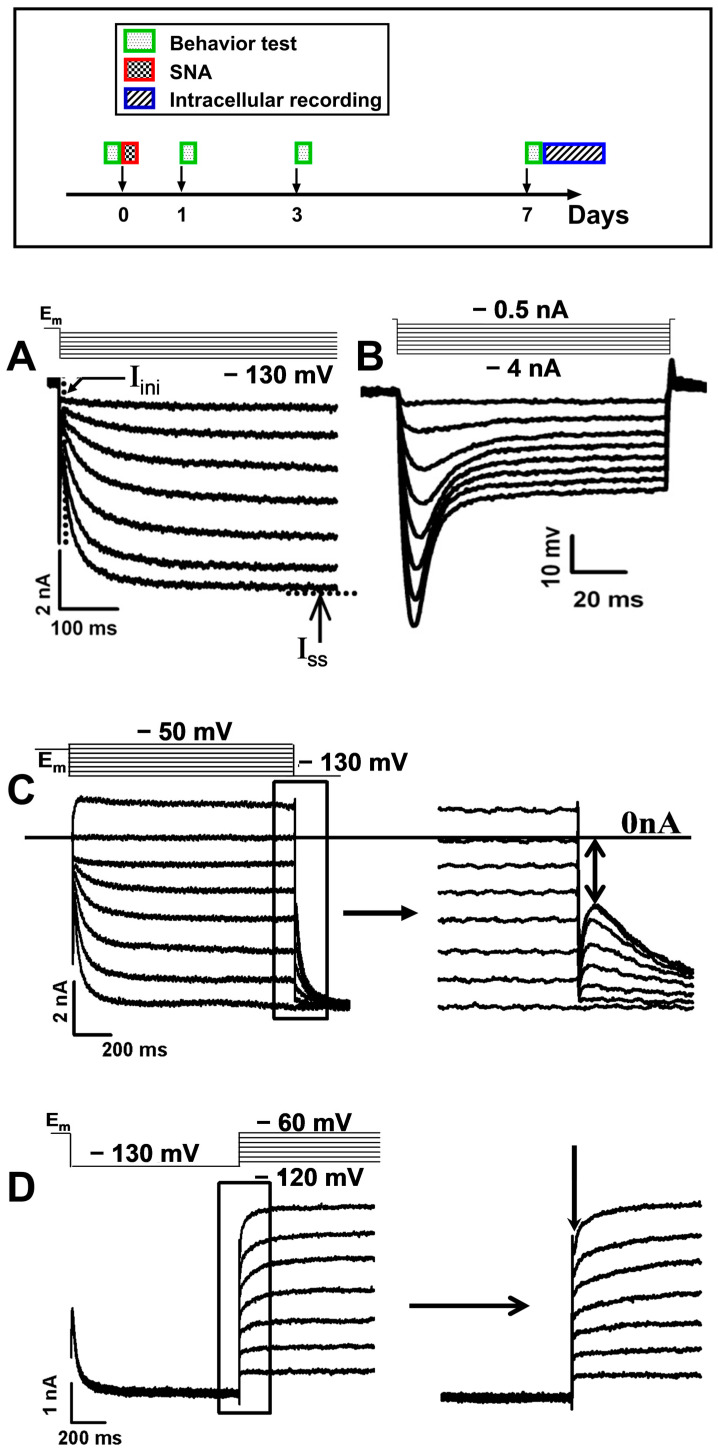
Experimental procedure and recording and measurement of I_h_ and kinetics. Upper panel, the time scale procedure for SNA, behavioral test, and intracellular recording. (**A**), the protocol and example of I_h_ recording by utilizing discontinuous single-electrode voltage clamp (d-SEVC) technique. I_h_ amplitude was measured as the difference between I_ss_ and I_ini_ (as arrow shown in the traces); (**B**), the depolarizing sag recorded under discontinuous current clamp (DCC) mode; (**C**), the deactivating current traces for measurements of reversal potential (V_rev_) and neuronal conductance (G_h_); and (**D**), the activating current traces for measurement of half activation potential (V_0.5_).

The L5 SNA model was employed in this study. The following procedures were conducted (Figure 6, upper panel): (1) Tactile allodynia, cold allodynia, and thermal hyperalgesia were assessed prior to SNA and on days one, three, and seven post-SNA; (2) intracellular action potential (AP) recordings and in vivo I_h_ recordings were performed in L5 DRG neurons of normal rats and rats after seven days of SNA; (3) HCN1 immunocytochemical images were compiled and analyzed; (4) ABC immunocytochemistry was utilized to evaluate the distribution of HCN1-2 proteins in both ipsilateral and contralateral L5 DRG neurons; and (5) quantitative reverse transcription polymerase chain reaction (RT-PCR) was conducted to measure the levels of *HCN*1-4 mRNA relative to GAPDH in both ipsilateral and contralateral sides of the L5 DRGs.

Young adult female (6~8 weeks) Sprague–Dawley (SD) rats, weighing between 150 and 180 g, were maintained under standard conditions, which included unrestricted access to food and water and a natural light/dark cycle. The rats were euthanized via cervical dislocation, following approval from the university’s ethical committee.

### 4.1. Preparing Neuropathic Pain Model: SNA Model

Tight ligation followed by axotomy of the left L5 spinal nerve was performed under sterile and aseptic conditions. In brief, the animals were deeply anesthetized using isoflurane (5% isoflurane mixed with oxygen) and positioned in a prone orientation. An incision was made at the midpoint of the spine at the L4-S1 level. The ventral ramus of the L5 spinal nerve was isolated, tightly ligated with a 6-0 silk suture (Ethicon, Brussels, Belgium; Look, Taunton, UK), and transected (L5 axotomy) just distal to the suture, ensuring that the spared L4 spinal nerve was not damaged. The skin incision was subsequently closed with two layers of sutures (subcutaneous and cutaneous).

### 4.2. Intracellular Recording

At day seven after SNA, an intracellular recording was performed in axotomized L5 DRG. Details of animal preparation is as described previously [32,33,34,35]. In brief, animals were deeply anaesthetized (sodium pentobarbital, 70–80 mg/kg, i.p.) with areflexia. A tracheotomy was performed to allow artificial ventilation and monitoring of end-tidal CO_2_. The right external jugular vein was cannulated to enable regular injections of additional doses of anesthetic (sodium pentobarbital, 10 mg/kg, i.v. hourly) which was required to maintain deep anesthesia. Blood pressure was monitored and maintained as stable throughout the experiments, indicating that the anesthesia was deep enough by applying the above doses of anesthetic. The axotomized L5 DRG on the left side was exposed and the corresponding dorsal root was sectioned close to the spinal cord and placed on a bipolar platinum electrode. The animal core temperature was maintained at 36 °C (±0.5), and the temperature in the paraffin pool was controlled within 28.5~32 °C throughout.

For the stability of recording, a small silver platform was placed beneath DRG, and muscle relaxant (pancuronium bromide, 0.6 mg/kg, i.v.) was administered intravenously through the jugular vein cannula just before recordings to block the response of muscles to electrical stimuli. The application of muscle relaxant was always accompanied by an additional dose of anesthetic (sodium pentobarbital, 10 mg/kg, i.v.) at regular interval (approximately hourly). Glass electrodes were filled with 3M KCl (40–90 MΩ). Cells were impaled by advancing the microelectrode in 1 µm step until a membrane potential was seen. A small capacitance buzz was usually applied at 10–20 μm distance interval to help penetrate the neuronal membrane. For each membrane potential obtained, the dorsal root was stimulated through bipolar electrode. Somatic action potentials (APs) were evoked with a single rectangular pulse (0.03 ms duration for A-fiber units and 0.3 ms for C-fiber units). The stimulus intensity was adjusted to twice threshold for A-fiber units and suprathreshold for C-fiber units.

### 4.3. Conduction Velocity (CV)

CV was determined from the conduction distance between the recorded neuron to the cathode divided by the latency, as described previously [36]. The latency was the time difference between the stimulus artefact and the beginning of the somatic AP without taking utilization time into account. Neurons were classified as C-, Aδ-, and Aα/β-fiber units while the CVs of the AP were in the range of <1.0, 1.5~6.5, and >6.5 m/s, respectively [32].

### 4.4. In Vivo I_h_ Recording

For I_h_ recording, the glass microelectrode contained 3M KCl (40–90 MΩ). After AP recording and identification of sensory properties, discontinuous single-electrode voltage clamp (dSEVC) was performed to record soma I_h_, after carefully balancing the bridge and neutralizing electrode capacitance. Neurons were held at resting E_m_ if it was in the −50 to −60 mV range, otherwise they were held at −60 mV and hyperpolarized from holding potential to −130 mV at the increments of −10 mV for one sec. I_h_ and I_h_ density were compared at −50 and −60 mV holding potentials and were unaffected within this range (*p* > 0.2, n = 6). Data were sampled at 8 kHz by an Axo-clamp 200A amplifier and filtered at 1 kHz.

### 4.5. I_h_, Cell Capacitance, and I_h_ Density

I_h_ consisted of an instantaneous inward current (I_ins_) and a steady-state current (I_ss_) (see Figure 1A). At each testing potential, I_h_ was measured as the difference between I_ss_ and I_ins_. More than two thirds of neurons had a clear “notch” that indicated the initiation of I_h_ activation. In other neurons, we measured the current 12 to 15 ms after the start of each voltage command to avoid the influence of capacitive artifacts [37]. As relatively high series resistance in sharp electrode recording affected the testing potentials, they differed slightly from the command potentials. To compare I_h_ between neurons, I_h_ at −100 mV was obtained by fitting original I_h_ versus testing potential plots with the Boltzmann equation (r^2^ > 99.99%) as follows: I_h_ = I_h0_ + I_hm_/(1 + exp^−((V−V^_0.5_^)/k)^), where I_h_ was I_h_ amplitude recorded at testing potential, I_h0_ was the fitted minimal I_h_, I_hm_ was the fitted maximal I_h_, V was testing potential, V_0.5_ was potential for half activation of I_h_, and K was the slope factor. I_h_ in this paper, from this point onwards, refers to I_h_ read at −100 mV from graphs of the Boltzmann equation.

Under discontinuous current clamp (DCC) mode, a series of negative current injections (−0.5 to −4 nA with a step of −0.5 nA, 100 ms duration) was applied to I_h_-expressing neurons to evoke a series of voltage responses (see Figure 1B). Between 20% and 80% of the potential change from its onset was fitted by an exponential decay function to yield input resistance (*R_i_*) and membrane time constant (*τ_m_*). Cell capacitance (*C_i_*) was calculated from *R_i_* divided by *τ_m_*. The final *C_i_* was the averaged cell capacitances from all the testing current injections.

I_h_ density at −100 mV for each neuron was then calculated from I_h_ at −100 mV divided by cell capacitance.

### 4.6. I–V Relationship and Reversal Potential (V_rev_)

I_h_ was fully activated by a prepulse at −130 mV for 1 s and then deactivated by a series of depolarization steps from −120 to −60 mV with an increment of −10 mV, duration one sec (see Figure 1C). The tail currents at the end of the prepulse, the downwards arrows shown in Figure 1C, were measured and plotted against testing potentials. The best fit I–V relationship was then determined using linear regression analysis and the equation I_tail_ = G_h_ (V_m_ − V_rev_), inputting I_tail_ and V_m_ (the testing potential) to find the best fit (r^2^ > 99%). This provided G_h_ (the neuronal conductance) and V_rev_ (the reversal potential of I_h_).

### 4.7. Half-Activation Potential (V_0.5_)

The activation potential was calculated from instantaneous tail currents generated by switching to −130 mV for 1 s depolarizing prepulses with an increment of −10 mV from −60 to −130 mV, with an increment of −10 mV (see Figure 1D). The instantaneous tail currents at −130 mV were measured, normalized to the maximal one (obtained with the −130 mV prepulse), and plotted against corresponding prepulses to yield activation curves (see Figure 1D).

The half-activation potential (V_0.5_) was obtained by fitting the curve by a Boltzmann equation of the following form: I_tail_/I_tail(max)_ = (1 + e((V_m_ − V_0.5_)/κ)) − 1, where V_m_ was the prepulse potential, I_tail_ was the instantaneous tail current recorded immediately after the prepulse, and I_tail(max)_ was the tail current after a prepulse of −130 mV (Figure 1D). This provided V_0.5_ was the potential at which I_h_ was half-activated, and κ was the slope factor.

### 4.8. Time Constant for I_h_ Activation (τ)

The time constant refers to the time that the system takes to reach approximately 63% of its final value. In this study, the time constant of activation was yielded by fitting current traces (Figure 1A) in each voltage step by a single exponential equation (r^2^ > 0.99 for all) as follows: *I_t_* = *I*_ss_ + *I*_h_·exp − *t*/τ, where *I_t_* was the current at time *t*, *I*_ss_ was the steady-state current, I_h_ was the amplitude of I_h_ at the corresponding testing potential, and *τ* was the time constant for *I*_h_ activation. This provides τ (the time constant for I_h_ activation) for each testing potential. For each neuron, τ was plotted against all testing potentials and fitted the graph (using the exponential growth equation). This enabled τ to be read off for each cell at standard testing potentials (from −70 to −120 at 10 mV intervals).

### 4.9. Behavior Testing

All experimental rats maintained good health and showed levels of exploratory activity, feeding activity, and weight gain indistinguishable from those of the unoperated normal rats. Responses were taken one day prior to surgery and at one, three, and seven days postoperatively.

The rats were placed singly in a clear plastic chamber (22 cm × 18 cm × 14 cm) on a glass floor at room temperature and, after 10 min for acclimatization, their postures for standing, walking, and resting were monitored daily up to day seven post operation.

Behavioral signs thought to represent stimulus-evoked neuropathic pain were studied as follows: (1) Mechanical allodynia (hypersensitivity to a normally innocuous stimulus) was indicated by withdrawal thresholds to innocuous von Frey hairs [38]. (2) Cold allodynia (hypersensitivity to cold stimulus) was indicated by response of flinching or paw shaking to application of acetone [39]. (3) Heat hyperalgesia was indicated by latency of withdrawal from a noxious heat stimulus (Hargreaves analgesymeter) [40].

### 4.10. Von Frey Hair Withdrawal Threshold (Mechanical Allodynia)

Each rat was placed on a metal mesh floor. The plantar surface of each hind paw was touched for ~2 s with a series of ten von Frey filaments (Touch-Test; North Coast Medical, Morgan Hill, CA) providing forces of 0.6, 1, 1.4, 2, 4, 6, 8, 10, 15, and 26 g. The diameters of these filaments are 0.20, 0.23, 0.25, 0.31, 0.36, 0.38, 0.41, 0.43, 0.48, and 0.56 mm, respectively. Each filament was applied totally five times to different place of the middle of plantar surface until 100% withdrawal was achieved from the five applications [38]. A minute was allowed between tests on alternate hind paws and two min between subsequent tests on the same hind paw. A positive response was recorded if the paw was withdrawn during the application of the von Frey filament, or immediately after its removal. The forces that can evoke a positive response (withdrawal) in the contralateral foot and in normal untreated rats were >2.0 g in all animals and >4.0 g in some animals. Thus, responses to a force <2.0 g are considered to be allodynic, because they do not evoke a positive response in normal rats or contralateral hindlimbs. The 50% withdrawal threshold was then determined from the stimulus–response curve generated.

### 4.11. Acetone Test (Cold Allodynia)

Each rat was placed on a metal mesh floor. A drop of acetone from a one ml syringe was applied directly to the plantar surface of each hind paw. In total, five rounds of acetone were applied to each side with three min interval between each application. A flinching, licking, or paw shaking response during or just after acetone application was considered to be positive and arbitrarily scored as one. No response would be scored as zero.

### 4.12. Withdrawal Latency to a Noxious Heat Stimulus (Heat Hyperalgesia)

A Plantar (Hargreaves) analgesymeter (Ugo Basile, Comerio, Italy) [41] was used to measure the latency of response to a noxious heat stimulus applied to the plantar surface of the hind paw. Each rat was placed on a 2 mm thick glass floor under which the laser radiant heat source was positioned. The stimulus onset activated a timer that was automatically stopped when the evoked paw withdrawal was detected by a photocell. Three latency measurements were taken and averaged for each hind paw during each session of testing. A reduced response latency to a normally noxious heat stimulus is considered to represent hyperalgesia.

The hind paws were tested alternately with at least five min intervals between consecutive stimuli on the same hind paw.

### 4.13. Quantitative RT- PCR

Real-time PCR was performed on L5 DRGs in ipsi and contra-lateral side at seven days after SNA by using SYBR Green detection (BioRad, Hercules, CA, USA). cDNA template was made from 5 μg total RNA using the Omniscript Reverse Transcription Kit (Qiagen, Shenzhen, China, catalog no.: 205111). The PCR reactions contained 0.3 μL SYBR green I MaxtMix (Eurogentec, Seraing, Belgium, catalog no.: RT-SN2X-03T), 1 μL cDNA template, one μL each of the 10X forward and reverse primers (200 nM), and 2.7 μL water. The conditions were 95 °C for 15 min followed by 40 cycles of denaturing at 95 °C for 30 s, annealing at 60 °C for 20 s, and extension at 72 °C for 40 s. Melt curves were also analyzed to ensure only one product per well. Primer sequences were either purchased from Qiagen or designed with the software Primer Express 2.0 (Applied Biosystems). All the primers were designed to span exon/exon boundaries to avoid co-amplification of possible contaminated genomic DNA. The specificity of the designed primers was checked with BLAST (http://www.ncbi.nlm.nih.gov/BLAST, accessed on 1 December 2014) and then ordered from MWG (Eurofins MWG Operon). Primer sequences and GenBank accession numbers for the genes selected for PCR validation are listed in Table 1. Each reaction was completed in triplicate wells on one plate, and each plate was run in triplicate. GAPDH was used as the endogenous reference gene for all the transcripts tested in these experiments.

Fold change between ipsilateral and contralateral L5 DRGs was calculated with the comparative CT method.

### 4.14. Histological Procedures

At the end of recording, rats under very deep anesthesia were perfused through the heart with 0.9% saline and then Zamboni’s fixative. DRGs were removed and post-fixed for 60 min in Zamboni’s solution and then stored overnight at 4 °C in 30% sucrose buffer. Serial 7 μm cryostat sections were cut and collected on 20 gelatin-coated slides. Tissue sections from both ipsi- (in the left row) and contra-lateral sides (in the right row) of DRG were collected at approximately 400 µm intervals. Thus, sections in each slide included the very beginning, the middle part, and the end of the DRGs. Slides were stored at −20 °C until immunocytochemistry was performed [42].

Before immunocytochemistry, endogenous peroxidase and biotin-like activity were blocked with 3% H_2_O_2_ and an avidin–biotin blocking kit (Vector Laboratories, Peterborough, UK), respectively. Then sections were incubated for 30 min with 10% normal goat serum (NGS) in PBS. ABC Immunocytochemistry was performed with an ABC kit (Vector Laboratories). The details of ABC immunocytochemistry procedure were described previously [42]. Briefly, sections were incubated overnight at 4 °C in primary antibody (HCN1, 1:1000; HCN2, 1:1000) in 0.05% Triton X-100 in PBS buffer with 1% NGS. Primary antibody was washed off and sections were then incubated for 30 min with biotinylated secondary antibody (anti-rabbit IgG, 1:200; Vector Laboratories). After washing with PBS/Triton, 3, 3′-diaminobenzidine (DAB) was used to form a color reaction product. Sections were dehydrated and mounted with carbonate glycerol buffer, cover-slipped, and sealed with nail varnish. No staining was seen when the procedure described above was used, but the primary antibody was omitted.

### 4.15. Primary Antibodies and Antibody Characterization

Anti-HCN1 primary antibody (Alomone, Jerusalem, Israel, 1:1000) was used for image analysis of immunoreactivity, and both anti-HCN1 and anti-HCN2 (Alomone company, 1:2000) were used for general image comparison. Antibody characterization was described in previous paper [10].

The anti-HCN1 rabbit polyclonal antibodies against N-terminus peptides 6-24 was from Alomone Labs Ltd., Jerusalem, Israel. This had been characterized with Western Blots and showed the same staining characteristics as an antibody raised in guinea pig against the C terminus of rat HCN1 [43]. Furthermore, HCN1 channel was selectively expressed in COS7 or HEK cells, and the specificity of the antibody has been confirmed by Western blots. The secondary antibodies were biotinylated anti-rabbit IgG (1:200) from Vector Laboratories, Peterborough.

### 4.16. Image Capturing and HCN1 Analysis

Sections were scanned using a Leica (Milton Keynes, UK) DMRBE light microscope and montaged using Simple PCI software 6.6 (Digital Pixel, Brighton, UK). The light settings on the microscope were kept identical for the montaging of all sections. For every montage, the illumination was adjusted to level seven on the microscope, and within the Simple PCI settings, the offset (black) was set at −25 with the gain at 1.85. One 7 μm section was collected every 400 μm through the DRG. For a 2 mm long DRG, there are normally five sections in the series. Sections one, three, and five were montaged under x40 illumination using the automatic stage control driven by the PCI software for ipsilateral and contralateral L5 DRGs. Any unevenness in background illumination was automatically digitally removed before montaging. Sections one, three, and five were analyzed, but if any of these was missing or damaged, an adjacent section was used.

The average pixel density for HCN1 staining in the montaged sections was measured using Simple PCI. All neurons with visible nuclei within the above sections were measured. Both membrane-associated (ring) and cytoplasmic intensity were measured by drawing three circles: one over the neuronal membrane, one inside the ring, and one around the nucleus. The following variables were provided from the measurements neuronal size and average pixel density both in cytoplasm and in the ring. The average pixel density (maximum range 0~255) was related to the intensity of HCN1 staining in the neuron. Every neuron with a visible ring was measured in this way; whereas in neurons without a visible ring, the second circle was drawn just inside the first circle marking the cell perimeter. To avoid bias during the measurement, sections were measured in a random order.

### 4.17. Criteria for Data Acceptance

Data selected for analysis fulfilled the following criteria: (1) E_m_ was stable, i.e., varied < 5 mV, during I_h_ recording; (2) E_m_ was −40 mV or more negative; (3) AP amplitude was ≥40 mV; and (4) APs were overshooting or had peak not lower than −10 mV for Aα/β-neurons, since some MSAs normally have non-overshooting somatic APs [33].

### 4.18. Statistics

Data were examined to determine whether they were normally distributed (GraphPad software Prism 10, San Diego, CA, USA). If not, non-parametric tests, Kruskal–Wallis test for comparison of more than two medians and Mann–Whitney U tests for comparison of two medians, were performed. Tests were two-tailed and a level of *p* < 0.05 was considered to be statistically significant. For correlation tests, either non-parametric Spearman correlation or linear regression were used, according to the distribution of those data.

## 5. Conclusions

The current in vivo findings indicate that I_h_ expression in DRG neurons is reduced and becomes more difficult to activate seven days following spinal nerve axotomy. This observation implies that I_h_ is unlikely to function as a “pacemaker” driving neuropathic pain, thereby suggesting that strategies aimed at reducing or blocking I_h_ may not be appropriate for the treatment of neuropathic pain.

## Figures and Tables

**Table 1:** Primers for *HCN*1-4 and GAPDH.

Encoding Protein	Genebank no.	Primer Sequence (All From 5′ to 3′)	Concentration (µM)	Span Exon/Exon
*HCN*1		Cat. no. QT00184674 (Qiagen)	200	NO
*HCN*2		GCGTGCCTTTGAGACCGT (F)	200	YES
		TTCTTGCCTATGCGGTCCA (R)		
*HCN*3		GGCAAAAAGAATTCGATATTGCA (F)	200	YES
		GCCTGGACTCGGCTCAGAG (R)		
*HCN*4		TCTGGACCGCATAGGCAAG (F)	200	YES
		CCTGAGTTGAGGTCGTGCTG (R)		
GAPDH		CCCTGTTCTAGAGACAGCCGC (F)	2	YES
		TGAGACGAGGCTGGCACTG (R)		

## Data Availability

The original contributions presented in this study are included in the article. Further inquiries can be directed to the corresponding author.

## References

[1] Baron R., Binder A., Wasner G. (2010). Neuropathic pain: Diagnosis, pathophysiological mechanisms, and treatment. Lancet Neurol..

[2] Santello M., Nevian T. (2015). Dysfunction of cortical dendritic integration in neuropathic pain reversed by serotoninergic neuromodulation. Neuron.

[3] Santello M., Bisco A., Nevian N.E., Lacivita E., Leopoldo M., Nevian T. (2017). The brain-penetrant 5-HT(7) receptor agonist LP-211 reduces the sensory and affective components of neuropathic pain. Neurobiol. Dis..

[4] Chaplan S.R., Guo H.Q., Lee D.H., Luo L., Liu C., Kuei C., Velumian A.A., Butler M.P., Brown S.M., Dubin A.E. (2003). Neuronal hyperpolarization-activated pacemaker channels drive neuropathic pain. J. Neurosci..

[5] Liu C.N., Michaelis M., Amir R., Devor M. (2000). Spinal nerve injury enhances subthreshold membrane potential oscillations in DRG neurons: Relation to neuropathic pain. J. Neurophysiol..

[6] Liu X., Chung K., Chung J.M. (1999). Ectopic discharges and adrenergic sensitivity of sensory neurons after spinal nerve injury. Brain Res..

[7] Sun Q., Xing G.G., Tu H.Y., Han J.S., Wan Y. (2005). Inhibition of hyperpolarization-activated current by ZD7288 suppresses ectopic discharges of injured dorsal root ganglion neurons in a rat model of neuropathic pain. Brain Res..

[8] He J.T., Li X.Y., Zhao X., Liu X. (2019). Hyperpolarization-activated and cyclic nucleotide-gated channel proteins as emerging new targets in neuropathic pain. Rev. Neurosci..

[9] Lee D.H., Chang L., Sorkin L.S., Chaplan S.R. (2005). Hyperpolarization-activated, cation-nonselective, cyclic nucleotide-modulated channel blockade alleviates mechanical allodynia and suppresses ectopic discharge in spinal nerve ligated rats. J. Pain.

[10] Gao L.L., McMullan S., Djouhri L., Acosta C., Harper A.A., Lawson S.N. (2012). Expression and properties of hyperpolarization-activated current in rat dorsal root ganglion neurons with known sensory function. J. Physiol..

[11] Djouhri L., Koutsikou S., Fang X., McMullan S., Lawson S.N. (2006). Spontaneous pain, both neuropathic and inflammatory, is related to frequency of spontaneous firing in intact C-fiber nociceptors. J. Neurosci..

[12] Horn S., Quasthoff S., Grafe P., Bostock H., Renner R., Schrank B. (1996). Abnormal axonal inward rectification in diabetic neuropathy. Muscle. Nerve..

[13] Yang Q., Kaji R., Takagi T., Kohara N., Murase N., Yamada Y., Seino Y., Bostock H. (2001). Abnormal axonal inward rectifier in streptozocin-induced experimental diabetic neuropathy. Brain..

[14] Yao H., Donnelly D.F., Ma C., LaMotte R.H. (2003). Upregulation of the hyperpolarization-activated cation current after chronic compression of the dorsal root ganglion. J. Neurosci..

[15] Tu H., Deng L., Sun Q., Yao L., Han J.S., Wan Y. (2004). Hyperpolarization-activated, cyclic nucleotide-gated cation channels: Roles in the differential electrophysiological properties of rat primary afferent neurons. J. Neurosci. Res..

[16] Song Y., Zhang M., Tao X., Xu Z., Zheng Y., Zhu M., Zhang L., Qiao J., Gao L. (2018). Difference of acute dissociation and 1-day culture on the electrophysiological properties of rat dorsal root ganglion neurons. J. Physiol. Biochem..

[17] Kouranova E.V., Strassle B.W., Ring R.H., Bowlby M.R., Vasilyev D.V. (2008). Hyperpolarization-activated cyclic nucleotide-gated channel mRNA and protein expression in large versus small diameter dorsal root ganglion neurons: Correlation with hyperpolarization-activated current gating. Neuroscience.

[18] Zhang X.F., Zhu C.Z., Thimmapaya R., Choi W.S., Honore P., Scott V.E., Kroeger P.E., Sullivan J.P., Faltynek C.R., Gopalakrishnan M. (2004). Differential action potentials and firing patterns in injured and uninjured small dorsal root ganglion neurons after nerve injury. Brain Res..

[19] Weng X., Smith T., Sathish J., Djouhri L. (2012). Chronic inflammatory pain is associated with increased excitability and hyperpolarization-activated current (Ih) in C- but not Aδ-nociceptors. Pain.

[20] Djouhri L., Fang X., Koutsikou S., Lawson S.N. (2012). Partial nerve injury induces electrophysiological changes in conducting (uninjured) nociceptive and nonnociceptive DRG neurons: Possible relationships to aspects of peripheral neuropathic pain and paresthesias. Pain.

[21] Abdulla F.A., Smith P.A. (2001). Axotomy- and autotomy-induced changes in Ca^2+^ and K^+^ channel currents of rat dorsal root ganglion neurons. J. Neurophysiol..

[22] Song Y., Li H.M., Xie R.G., Yue Z.F., Song X.J., Hu S.J., Xing J.L. (2012). Evoked bursting in injured Aβ dorsal root ganglion neurons: A mechanism underlying tactile allodynia. Pain.

[23] Sartiani L., Mannaioni G., Masi A., Novella R.M., Cerbai E. (2017). The Hyperpolarization-Activated Cyclic Nucleotide-Gated Channels: From Biophysics to Pharmacology of a Unique Family of Ion Channels. Pharmacol. Rev..

[24] Sánchez-Alonso J.L., Halliwell J.V., Colino A. (2008). ZD 7288 inhibits T-type calcium current in rat hippocampal pyramidal cells. Neurosci. Lett..

[25] Lees-Miller J.P., Guo J., Wang Y., Perissinotti L.L., Noskov S.Y., Duff H.J. (2015). Ivabradine prolongs phase 3 of cardiac repolarization and blocks the hERG1 (KCNH2) current over a concentration-range overlapping with that required to block HCN4. J. Mol. Cell. Cardiol..

[26] Melgari D., Brack K.E., Zhang C., Zhang Y., El H.A., Mitcheson J.S., Dempsey C.E., Ng G.A., Hancox J.C. (2015). hERG potassium channel blockade by the HCN channel inhibitor bradycardic agent ivabradine. J. Am. Heart Assoc..

[27] Peters C.H., Liu P.W., Morotti S., Gantz S.C., Grandi E., Bean B.P., Proenza C. (2021). Bidirectional flow of the funny current (I(f)) during the pacemaking cycle in murine sinoatrial node myocytes. Proc. Natl. Acad. Sci. USA.

[28] Haechl N., Ebner J., Hilber K., Todt H., Koenig X. (2019). Pharmacological Profile of the Bradycardic Agent Ivabradine on Human Cardiac Ion Channels. Cell Physiol. Biochem..

[29] Vasylyev D.V., Liu S., Waxman S.G. (2023). I(h) current stabilizes excitability in rodent DRG neurons and reverses hyperexcitability in a nociceptive neuron model of inherited neuropathic pain. J. Physiol..

[30] Tanaka S., Ishida T., Ishida K., Fuseya S., Ito M., Sakamoto A., Kawamata M. (2022). A randomized, double-blinded, placebo-controlled, crossover study of the HCN channel blocker ivabradine in a capsaicin-induced pain model in healthy volunteers. Sci. Rep..

[31] Lee M.C., Bond S., Wheeler D., Scholtes I., Armstrong G., McNaughton P., Menon D. (2019). A randomised, double-blind, placebo-controlled crossover trial of the influence of the HCN channel blocker ivabradine in a healthy volunteer pain model: An enriched population trial. Pain.

[32] Fang X., Djouhri L., Black J.A., Dib-Hajj S.D., Waxman S.G., Lawson S.N. (2002). The presence and role of the tetrodotoxin-resistant sodium channel Na(v)1.9 (NaN) in nociceptive primary afferent neurons. J. Neurosci..

[33] Fang X., McMullan S., Lawson S.N., Djouhri L. (2005). Electrophysiological differences between nociceptive and non-nociceptive dorsal root ganglion neurones in the rat in vivo. J. Physiol..

[34] Fang X., Djouhri L., McMullan S., Berry C., Waxman S.G., Okuse K., Lawson S.N. (2006). Intense isolectin-B4 binding in rat dorsal root ganglion neurons distinguishes C-fiber nociceptors with broad action potentials and high Nav1.9 expression. J. Neurosci..

[35] Djouhri L., Fang X., Okuse K., Wood J.N., Berry C.M., Lawson S.N. (2003). The TTX-resistant sodium channel Nav1.8 (SNS/PN3): Expression and correlation with membrane properties in rat nociceptive primary afferent neurons. J. Physiol..

[36] Djouhri L., Lawson S.N. (2001). Increased conduction velocity of nociceptive primary afferent neurons during unilateral hindlimb inflammation in the anaesthetised guinea-pig. Neuroscience.

[37] Rodrigues A.R., Oertel D. (2006). Hyperpolarization-activated currents regulate excitability in stellate cells of the mammalian ventral cochlear nucleus. J. Neurophysiol..

[38] Chaplan S.R., Pogrel J.W., Yaksh T.L. (1994). Role of voltage-dependent calcium channel subtypes in experimental tactile allodynia. J. Pharmacol. Exp. Ther..

[39] Hulse R., Wynick D., Donaldson L.F. (2010). Intact cutaneous C fibre afferent properties in mechanical and cold neuropathic allodynia. Eur. J. Pain.

[40] Burdette B.H., Gale E.N. (1988). Pain as a learned response: A review of behavioral factors in chronic pain. J. Am. Dent. Assoc..

[41] Hargreaves K., Dubner R., Brown F., Flores C., Joris J. (1988). A new and sensitive method for measuring thermal nociception in cutaneous hyperalgesia. Pain.

[42] Lawson S.N., Crepps B.A., Perl E.R. (1997). Relationship of substance P to afferent characteristics of dorsal root ganglion neurones in guinea-pig. J. Physiol..

[43] Crunelli V., David F., Morais T.P., Lorincz M.L. (2023). HCN channels and absence seizures. Neurobiol. Dis..

